# Heterogeneous Skeletal Muscle Cell and Nucleus Populations Identified by Single-Cell and Single-Nucleus Resolution Transcriptome Assays

**DOI:** 10.3389/fgene.2022.835099

**Published:** 2022-05-13

**Authors:** Katherine Williams, Kyoko Yokomori, Ali Mortazavi

**Affiliations:** ^1^ Department of Developmental and Cell Biology, University of California, Irvine, Irvine, CA, United States; ^2^ Center for Complex Biological Systems, University of California, Irvine, Irvine, CA, United States; ^3^ Department of Biological Chemistry, School of Medicine, University of California, Irvine, Irvine, CA, United States

**Keywords:** single-cell RNA-seq, single-nucleus RNA-seq, spatial transcriptomics, myonuclei heterogeneity, skeletal muscle

## Abstract

Single-cell RNA-seq (scRNA-seq) has revolutionized modern genomics, but the large size of myotubes and myofibers has restricted use of scRNA-seq in skeletal muscle. For the study of muscle, single-nucleus RNA-seq (snRNA-seq) has emerged not only as an alternative to scRNA-seq, but as a novel method providing valuable insights into multinucleated cells such as myofibers. Nuclei within myofibers specialize at junctions with other cell types such as motor neurons. Nuclear heterogeneity plays important roles in certain diseases such as muscular dystrophies. We survey current methods of high-throughput single cell and subcellular resolution transcriptomics, including single-cell and single-nucleus RNA-seq and spatial transcriptomics, applied to satellite cells, myoblasts, myotubes and myofibers. We summarize the major myonuclei subtypes identified in homeostatic and regenerating tissue including those specific to fiber type or at junctions with other cell types. Disease-specific nucleus populations were found in two muscular dystrophies, FSHD and Duchenne muscular dystrophy, demonstrating the importance of performing transcriptome studies at the single nucleus level in muscle.

## Background

**FIGURE 1:**
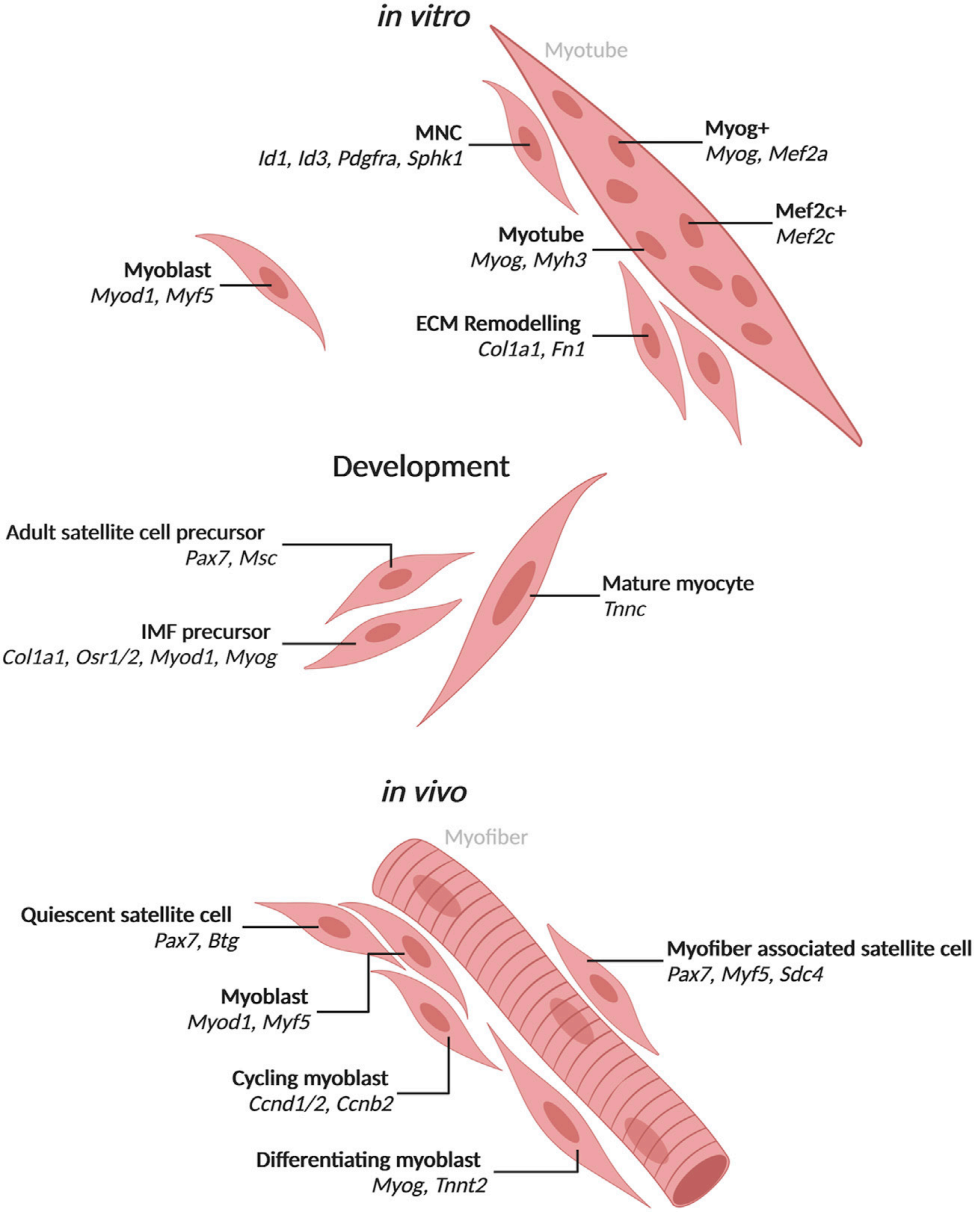
Populations of mononuclear skeletal muscle cells and myotube nuclei with associated markers identified by single-cell and single-nucleus RNA-seq Populations and top markers of cells and nuclei from mononucleated cells or myotubes. *In vitro* populations include cell and nuclear populations identified from *in vitro culture* of biopsy derived cells or muscle cell lines. Development populations include those observed during development in mice. *In vivo* populations include those observed in human or mouse muscle. Created with BioRender.com.

Skeletal muscle is the most abundant tissue in our bodies and is crucial for voluntary movement and support. Adult skeletal muscle tissue is composed primarily of mature muscle cells called myofibers and undifferentiated muscle cells called satellite cells. Myofibers can reach up to 30 cm in length in humans and 10 mm in mice and have hundreds of nuclei (Konno and Suzuki, 2000; Griffin et al., 1971). Muscle differentiation and fusion, called myogenesis, is controlled by a gene regulatory network well studied in mice (Blais, 2015; Chal and Pourquié, 2017). Skeletal muscle is initially specified in embryonic development when the somite segments into the dermomyotome, and muscle specification begins with the expression of transcriptional regulators Pax3 and Myf5 around embryonic day 9 (E9) in mouse (Chal and Pourquié, 2017). The muscle regulatory factors (MRFs) Myf5, Myod, Mrf4 and Myog control the transition from cycling to postmitotic cells that go on to form primary fibers (Chal and Pourquié, 2017). After the formation of these primary fibers, remaining cycling cells downregulate Pax3 and upregulate Pax7 (Chal and Pourquié, 2017). These cells either fuse to each other forming new myotubes or to the primary fibers by turning on the transcription factor Myod and then Myog (Chal and Pourquié, 2017). Pax7+ cells that do not fuse will continue to cycle and become satellite cells (Figure 1) (Chal and Pourquié, 2017). Satellite cells become activated in embryogenesis by turning on Myod and turning off Pax7 to form myoblasts which fuse to existing myofibers (Relaix and Zammit, 2012; Chal and Pourquié, 2017). Following Myog expression at day E11.5 in mouse, Mrf4 (herculin/Myf6) is turned on at day E13.5 and controls final myofiber structure such as myonuclear positioning (Huang, 2017). The major muscle specification and patterning is complete at this point (Huang, 2017). Myoblasts continue to fuse to myofibers after birth to build the muscle until postnatal day 21 in mice (Relaix and Zammit, 2012). At this point, the satellite cells also become quiescent, and muscle structure is established (Relaix and Zammit, 2012; Chal and Pourquié, 2017). Several myofibers group together to form fascicles, and multiple fascicles make up the total muscle (Jorgenson et al., 2020). Each individual myofiber is surrounded by a matrix of connective proteins termed the basal lamina (Schiaffino and Reggiani, 2011). Satellite cells reside under the basal lamina in direct contact with the myofiber (Chal and Pourquié, 2017).

Myogenesis in adult muscle follows a similar trajectory as seen in development. Regeneration is stimulated upon injury when satellite cells become activated (Zanou and Gailly, 2013). For both mice and humans, quiescent satellite cells expressing Pax7 become activated, express Myod and proliferate (Figure 1) (Zanou and Gailly, 2013). Some myoblasts continue to proliferate while others commit to differentiation (Relaix and Zammit, 2012). Differentiating myoblasts turn on Myog after exiting the cell cycle such that early myotubes are marked by Myog expression (Figure 1) (Relaix and Zammit, 2012), which is important for activation of numerous myogenesis genes and fusion (Zanou and Gailly, 2013). The cells can fuse to existing myofibers but more commonly fuse to each other to form nascent myotubes (early differentiated, postmitotic muscle cells) and eventually myofibers (Rochlin et al., 2010). Myog expression wanes and Mrf4 is expressed to control intracellular structure as in embryogenesis (Zanou and Gailly, 2013). Understanding regeneration is important to the study of muscular diseases such as dystrophies or sarcopenia (the loss of muscle with age) that involve defects in muscle repair (Robinson and Dilworth, 2018).

The myogenesis process up to myotube formation can be mimicked *in vitro* for human and mouse using primary myoblasts or stem cells isolated from tissue or from induced pluripotent stem cells (iPSCs) differentiated to form myoblasts. *In vitro* myoblasts are triggered to differentiate using serum or with ITS (insulin-transferrin-selectin) (van der Wal et al., 2018). Similar to *in vivo* differentiation, cells which activate Myod elongate and begin to fuse eventually turning on Myog (Figure 1). Myog + myotubes make up a majority of cells by 72 h post-induction with a number of mononuclear cells still present (Figure 1). Past 72 h, myotubes will continue to grow and add more nuclei, but these myotubes cannot form full myofibers in 2D culture. A number of studies have worked on 3D culture to form something more akin to myofibers (Jones et al., 2018). *In vitro* culture has provided an invaluable tool for studying skeletal muscle myogenesis. However, the systems lack interaction with other cell types such as neurons and tendons which are present in tissue and help to shape the muscle cells.

Neurons and tendons make direct contact with myofibers at specialized junctions in both mice and humans. The nuclei along the length of the myotube are able to respond to local signals at the junctions thereby making nuclei within a myofiber heterogenous in terms of incoming signals and transcriptional output (Wu et al., 2010). Neurons interact with muscle at the neuromuscular junction (NMJ). The nuclei clustered underneath the NMJ, called fundamental myonuclei, transcribe specialized genes such as *ACHE* (acetylcholinesterase), which hydrolyzes the acetylcholine signals from the motor neuron (Jacobson et al., 2001; Schiaffino and Reggiani, 2011; Castets et al., 2020). Tendons interact with muscle at the myotendinous junction (MTJ) where myonuclei express collagens including *Col22a1* (Charvet et al., 2013). The MTJ is crucial for skeletal muscle and tendon development as the tendon guides the muscle to attach and muscle contraction maintains the tendon cells (Subramanian and Schilling, 2015). Myonuclei are therefore specialized within the myofiber.

The transcriptome of skeletal muscle has been relatively well studied from *in vitro* to *in vivo* systems. Several studies have assessed the transcriptome of muscle cell lines and biopsies using microarray and RNA sequencing (RNA-seq) from mouse and human (Blais, 2015). These studies have provided valuable insights into myogenesis. For example, RNA-seq on human iPSC-derived skeletal muscle cells found TWIST1 to be important for maintaining *PAX7* expression in satellite cells (Choi et al., 2020). Additionally, the transcription factor Tead4 was found to regulate differentiation in the mouse skeletal myoblast line C2C12 (Benhaddou et al., 2012). Transcriptome studies have been especially valuable for disease studies such as muscular dystrophies and sarcopenia (Su et al., 2015; Gonorazky et al., 2016; Jagannathan et al., 2016). These studies have been crucial to our understanding of skeletal muscle biology and molecular mechanisms of disease. However, these studies have been limited in their findings due to limitations of profiling multiple heterogenous cells together. Pooling of cells in different states of differentiation for example can lead to averaging of expression from subsets of cells. To identify the timing of MRF expression, satellite cells were isolated by hand to avoid pooling cells in different states of myogenesis (Cornelison and Wold, 1997). Recent advances in transcriptomics have enabled high-throughput single-cell and single-nucleus RNA-seq and spatial transcriptomics to assay transcriptional heterogeneity at high resolution. In this review, we will go over current applications of high-throughput, high-resolution transcriptomic techniques to the different stages of skeletal muscle cell differentiation in human and mouse. We will cover their uses in understanding basic biology of muscle cells as well as application to development, regeneration, aging and disease.

### High-Resolution Transcriptome Methods

Single-cell RNA-seq (scRNA-seq) revolutionized the field of transcriptomics, and multiple studies have applied this to assay mononucleated satellite cells (Schaum et al., 2018; Giordani et al., 2019; Rubenstein et al., 2020). However, application of scRNA-seq to skeletal muscle is challenging due to the size of myotubes and myofibers as most methods of scRNA-seq involve microfluidics that restrict the input cell size (10X Genomics, 2018). To assay these larger cell types, researchers have turned to hand-picking cells or to single-nucleus RNA-seq (snRNA-seq). Alternatively, spatial transcriptomics can be used to preserve the spatial context of the transcriptomic data. We will briefly discuss the advantages and disadvantages of these technologies for use in skeletal muscle (Table 1).

**TABLE 1:** Advantages and limitations of high-throughput transcriptome methods for skeletal muscle cell types.

	Mononucleated cells (Satellite cells and myoblasts)			Myotubes			Myofibers		
	scRNA-seq	snRNA-seq	Spatial transcript-omics	scRNA-seq	snRNA-seq	Spatial transcript-omics	scRNA-seq	snRNA-seq	Spatial transcript-omics
Muscle tissue	Yes^a^	Yes^a^	Yes	Yes^a^	Yes	Yes	Yes^b^	Yes	Yes^b^
Cell lines	Yes	Yes	Yes	Yes^c^	Yes	Yes	Yes^b^	Yes	Yes^b^
Throughput	High	High	Low to medium^d^	High	High	Low to medium^d^	Low	High	Low to medium^d^
Resolution	High	High	Low to high^d^	High	High	Low to high^d^	High	High	Low to high^d^
Assay multiple cell types at once	Yes	Yes	Yes	Yes	Yes	Yes	No	Yes	Yes
Obtain spatial expression information	No	No	Yes	No	No	Yes	No	No	Yes
Assay nuclear heterogeneity	Yes	Yes	Yes	No	Yes	Yes	No	Yes	Yes
Size restricted	Yes	No	No	Yes	No	No	Yes	No	No
Survey cytoplasmic transcripts	Yes	No	Yes	Yes	No	Yes	Yes	No	Yes
Associate nuclei with cell of origin	Yes	No	Yes	*NA*	No	Yes	*NA*	No	Yes
References	(Rubenstein et al., 2020; Giordani et al., 2019; Schaum et al., 2018; Kimmel et al., 2020; Dell’Orso et al., 2019; Zeng et al., 2016; Jiang et al., 2020; He et al., 2020; De Micheli et al., 2020a; Barruet et al., 2020; Hernando-Herraez et al., 2019)	(Zeng et al., 2016; Dos Santos et al., 2020; Kim et al., 2020; Petrany et al., 2020; Orchard et al., 2021)	(Kann and Krauss, 2019; Fomchenko et al., 2020; Petrany et al., 2020; McKellar et al., 2021)	(van den Heuvel et al., 2019; Fomchenko et al., 2020)	(Zeng et al., 2016; Jiang et al., 2020)	(Jiang et al., 2020; Chau et al., 2021)	Blackburn et al. (2019)	(Dos Santos et al., 2020; Kim et al., 2020; Petrany et al., 2020; Orchard et al., 2021)	(Kann and Krauss, 2019; Dos Santos et al., 2020; Fomchenko et al., 2020; Kim et al., 2020; Petrany et al., 2020; McKellar et al., 2021)

a Due to size restrictions or to isolate specific cell type, sample must be specially prepared by filtering or FACS, sorting.

b To survey the whole myofiber or fiber bundle (from 3D culture), fibers must be hand-picked.

c Due to size restrictions or to isolate specific cell type, sample must be specially prepared by filtering or FACS, sorting alone or in combination with fusion inhibition.

_d_ Throughput and resolution depend on the method used.

Single-cell RNA-seq (scRNA-seq) has already been widely used for studying mononucleated muscle cells (Saber et al., 2020). Both droplet and microfluidic based platforms have been used with sorted and unsorted mononucleated muscle cells from cell lines and satellite cells derived from tissues. This approach is relatively fast, and FACS sorting can help by filtering out low quality material such as debris, doublets and dead cells. Preparation of muscle stem cells, including FACS, can cause a stress induced transcriptome, which can be avoided with *in situ* fixation (Machado et al., 2017; van Velthoven et al., 2017). ScRNA-seq has been useful in understanding myogenesis in more detail as cells in different states of differentiation are distinguishable at single cell resolution (Kimmel et al., 2020; Shcherbina et al., 2020). ScRNA-seq from tissue has the advantage of capturing multiple cell types within a certain size. Analyzing multiple skeletal muscle resident cell types, such as fibroadipogenic progenitors (FAPs) or tenocytes, gives us a better understanding of interactions, perturbations and responses of the whole tissue (Rubenstein et al., 2020; Giordani et al., 2019; Dell’Orso et al., 2019). For mononuclear muscle cells, scRNA-seq is a fast, high-throughput and high-resolution way to profile the transcriptome.

Profiling the transcriptomes of mature muscle cells such as myotubes or myofibers can be done at the whole cell or nucleus level. Mature muscle cells can also be used for scRNA-seq, but isolation of individual cells has to be done manually due to their size which makes the method low throughput. Myofibers from a mouse can be anywhere from 2 to 10 mm in length (Griffin et al., 1971), while cultured differentiated myotubes can be over 100 μm in length (Jones et al., 2018). Most single-cell sequencing platforms can accommodate cells up to 60 μm (10X Genomics, 2018; Fluidigm, 2014; Illumina Inc., Bio-Rad Laboratories Inc, 2016). In order to use microfluidics based methods, some researchers have used single nucleated “myotubes” which are stimulated to differentiated, but fusion is blocked using a calcium chelator (van den Heuvel et al., 2019). These cells are smaller than myotubes and myofibers and therefore are easily captured on technologies such as the 10X chromium. Single-nucleus RNA-seq is a high-throughput alternative that involves lysing cells to isolate nuclei and to subsequently use them for RNA-seq. Single-nucleus RNA-seq has been widely used for neurons since their long and intricate morphologies can cause them to clump or be too large for scRNA-seq microfluidics systems (Krishnaswami et al., 2016; Habib et al., 2017; Lake et al., 2017; Bakken et al., 2018). However, the adaptation of snRNA-seq to other cell types, such as skeletal muscle, has been slow. Since the first application of snRNA-seq in muscle in 2016 (Zeng et al., 2016), only five papers have used snRNA-seq for skeletal muscle (Dos Santos et al., 2020; Jiang et al., 2020; Kim et al., 2020; Petrany et al., 2020; Orchard et al., 2021). Single-nucleus RNA-seq can be used for multiple cell types, for example isolated from a tissue, and is therefore able to profile both mononuclear and multinucleated cells together. For multinucleated cell types, snRNA-seq offers the additional advantage of resolving unicellular nuclear heterogeneity. This has proven useful for studies looking at transcriptomes of nuclei near neuromuscular junctions (NMJ) or myotendinous junctions (MTJ) (Dos Santos et al., 2020; Petrany et al., 2020). However, snRNA-seq in multinucleated cells has the distinct complexity that we cannot determine which nuclei originate from the same cell. Additionally, isolating the nucleus means only a fraction of the transcripts within a cell are surveyed, so nuclear resident transcripts such as long noncoding RNAs (lncRNAs) and pre-mRNA are enriched (Zeng et al., 2016). Nevertheless, snRNA-seq is a high-throughput, high-resolution method for transcriptome studies in mature skeletal muscle cell types.

Single-cell and nucleus RNA-seq have the important limitation that spatial information is lost as the cells or nuclei are removed from their native contexts for RNA isolation. The relative locations of cells and nuclei can be important for understanding cell type interactions and response. For example, the relative location of activated or quiescent satellite cells to pathological features such as fat deposits or immune infiltrates can be informative in disease contexts (Morgan et al., 2018; Wang et al., 2019). Myofiber nuclei are known to specialize based on their relative locations to non-muscle cell types (Kim et al., 2020; Petrany et al., 2020). For multinucleated cells, spatial transcriptomics has enabled the identification of transcriptionally distinct nuclei originating from the same myofiber (Kann and Krauss, 2019; Kim et al., 2020; Chau et al., 2021). This is an advantage no other method currently offers. Lower throughput *in situ* hybridization (ISH) methods have been used extensively in muscle to tag anywhere from one to four genes, such as with conventional RNA FISH and the original RNAscope (Chau et al., 2021). New medium throughput RNAscope methods are now available to assay tens of genes at a time (Phatak et al., 2019). High-throughput methods, such as seqFISH and multiplexed error-robust fluorescence ISH (MERFISH), have not been used in muscle, while 10X’s Visium platform has been used once (Chen et al., 2015; Eng et al., 2019; Marx, 2021; McKellar et al., 2021). A fine resolution trajectory of mouse muscle repair was generated by integrating numerous scRNA-seq studies with high-throughput spatial transcriptomics (McKellar et al., 2021). This work identified satellite cell activation with expression of *Myod1* near the injury site by 2 days after injury, and *Mymk* and *Mymx* activation around the injury 5 days after injury (McKellar et al., 2021). They also found potential paracrine signaling of *Mdk* from fibroadipogenic progenitors (FAPs) to nearby muscle cells that may be involved in injury repair (McKellar et al., 2021). Spatial transcriptomic methods have helped us understand how nuclei differ within a cell and how neighboring cells or environment affect transcriptional heterogeneity.

### Skeletal Muscle Cell and Nucleus Heterogeneity During Development

While muscle development during embryogenesis has been studied extensively, the resolution of single-cell RNA-seq can discern specific signals that may be confounded in bulk RNA-seq. During mouse embryogenesis, muscle cells are specified and differentiate into *Pax7*+ cells which will form satellite cells in the adult as well as myocytes that fuse to form myofibers (Chal and Pourquié, 2017). The satellite cell precursors express *Pax7* and its target *Msc* (Figure 1) (He et al., 2020). The mature myocytes express *Tnnc* (He et al., 2020). A set of cells in the embryonic limb bud express markers of both fibroblasts, *Col1a1* and *Osr1/2*, and muscle cells, *Myod1* and *Myog*, and may give rise to interstitial muscle fibroblasts (IMFs) which are able to differentiate to muscle (Figure 1; Supplementary Table S1) (He et al., 2020). This population would have been hidden in bulk RNA-seq studies due to the expression of markers from two cell types.

Heterogeneous populations of myonuclei were found in postnatal skeletal muscle in mice when muscle is still fusing. One population is found in the highest abundance in P21 mice when fusion stops (Petrany et al., 2020). It is marked by expression of *Myh9, Flnc* and possibly *Runx1*, *Nrap*, *Fhod3*, *Enah*, *Myh10*, *Ifrd1*, *Nfat5*, *Mef2a*, *Ell*, *Creb5*, *Zfp697* with no expression of the fiber type specific *Myh4* and *Myh1* (Dos Santos et al., 2020; Petrany et al., 2020). These nuclei are referred to as “sarcomere assembly states” due to the expression of genes related to “pre-myofibrils” used before mature myosins are in place (Petrany et al., 2020). The upregulation of the transcription factor *Atf3* and many of its target genes suggest a role for Atf3 in these myonuclei during development (Petrany et al., 2020). Two other distinct myonucleus populations are present in P21 mouse muscle marked by expression of *Meg3* or *Nos1* (Petrany et al., 2020). In developing tissue at P10 when cells are still fusing, myoblasts express *Myog* and *Mymk* which is crucial for fusion (Petrany et al., 2020). Some myonuclei appear to represent a transcriptional transition away from other cell types with expression of both myogenic markers *Ckm*, *Tnni2*, *Tnnt3* and ECM genes *Col1a1*, *Col3a1*, *Col5a3*, *Col6a1*, and *Dcn* (Petrany et al., 2020). These make up 4.7% of P10 myonuclei which are still developing and 0.8% of P21 myonuclei which have finished fusing (Petrany et al., 2020).

### Cell Populations Present During Skeletal Muscle Regeneration

Similar to development, *Pax7*-positive (*Pax7+*) satellite cells exit quiescence and become activated during adult skeletal muscle regeneration in mice and humans (Cornelison and Wold, 1997; Rochlin et al., 2010). They then proliferate with some cells retaining satellite cell identity while others differentiate to committed myoblasts that continue to divide. Myoblasts differentiate further to rebuild lost muscle (Rochlin et al., 2010). A number of studies have examined the regeneration of adult muscle cells and have identified *Pax7+* satellite cells in both mouse and human using scRNA-seq [Reviewed in (Saber et al., 2020)]. Satellite cells in quiescence express *Pax7* and *Btg2*, but upon activation express *Myod1* and *Myf5* as early activated cells (Dell’Orso et al., 2019; De Micheli et al., 2020a; De Micheli et al., 2020b; Oprescu et al., 2020). Of note, quiescent satellite cells transcribe *Myod1* but do not translate it until activated (de Morrée et al., 2017). When measured by RNAscope, 71% of mouse satellite cells attached to myofibers expressed *Myod1* (Kann and Krauss, 2019). Quiescent satellite cells that resist activation were found in human muscle marked by expression of *CAV1* (Barruet et al., 2020). After satellite cell activation, primary myoblasts express cell cycle related genes and can progress to one of two populations (Dell’Orso et al., 2019). Myoblasts that differentiate activate *Myog* and *Tnnt2*, while myoblasts that continue to proliferate express *Ccnd1/2* and *Ccnb2* (Dell’Orso et al., 2019; De Micheli et al., 2020a). Myoblast proliferation in mice is affected by interactions with the cell surface receptor family Syndecans (Sdcs) that are expressed in a subset of quiescent and cycling muscle cells (De Micheli et al., 2020a). *Sdc4* is expressed in 100% of quiescent satellite cells attached to myofibers when measured by RNAscope, while *Pax7* and *Myf5* were found in 99% of satellite cells (Kann and Krauss, 2019). Satellite cells found in injured muscle upregulate genes involved in processes such as glycolysis and the TCA cycle (Dell’Orso et al., 2019). Therefore, most but not all of the transcriptional heterogeneity in mononucleated muscle cells is attributable to myogenesis.

### Transcriptional Heterogeneity *in Vitro* Muscle Differentiation

Regeneration can be studied *in vitro* through differentiation of human myoblasts followed by scRNA-seq (Trapnell et al., 2014). *In vitro* differentiation is asynchronous with cells differentiating at different rates such that markers of myotubes are present in some cells as early as 24 h. Ordering cells by pseudotime arranged cells into a differentiation trajectory based on their transcriptome profiles rather than the time exposed to stimulus (Trapnell et al., 2014). *ID1* was found to have a switch-like inactivation, which is followed by activation of *MYOG* (Trapnell et al., 2014). *CUX1* and *USF1* were also identified as novel regulators of myogenesis (Trapnell et al., 2014). scRNA-seq was able to parse out signatures of differentiating myoblasts, while larger, more differentiated myotubes were assessed using snRNA-seq.

Single-nucleus RNA-seq in muscle cells was validated initially by comparing the transcriptomes of whole myoblasts to myoblast nuclei from *in vitro* culture (Zeng et al., 2016). Overall, the nuclear transcriptomes were found to faithfully recapitulate those of whole cells with the exception of enrichment for nuclear resident transcripts such as lncRNAs (Zeng et al., 2016). Single-nucleus RNA-seq of *in vitro* human myoblasts differentiated into myotubes for 72 h revealed a subset of nuclei expressing *ID1*, *ID3*, *PDGFRA* and *SPHK1* which appear mesenchymal (Trapnell et al., 2014; Zeng et al., 2016). These nuclei were from mononucleated muscle cells (MNCs) that failed to fuse, similar to the bifurcation of myoblast differentiation *in vivo* (Trapnell et al., 2014; Zeng et al., 2016). From mouse C2C12 culture differentiated for 72 h, snRNA-seq identified 8 clusters of nuclei that express *Pax7* and exhibit heterogeneity, which most likely represent unfused MNCs (Rebboah et al., 2021). Two of the 8 clusters represent proliferating cells with high expression of the cell cycle gene *Top2a* in one and *Lix1*, which is important for satellite cell proliferation, in the other (Rebboah et al., 2021). Another cluster of nuclei with significant expression of collagens and *Fn1* appears similar to a population of stem cells found *in vivo* that remodel their extracellular matrix and trigger proliferation in neighboring satellite cells (Bentzinger et al., 2013; Rebboah et al., 2021). This ECM cluster was distinct from another cluster that expressed *Itm2a* and *Pax7* and may be similar to activated satellite cells (Rebboah et al., 2021). Through RNAscope, *Myog* expression was detected in MNCs in addition to multinucleated myotubes (Rebboah et al., 2021). Nuclei from the most differentiated cells appear to either express *Myog* or *Mef2c* and could represent specialized myonuclei in culture (Rebboah et al., 2021). Nuclear heterogeneity in *vitro* culture provides evidence that myonuclei specialization is inherent and not solely due to contact with non-muscle cell types that is seen in tissue. *In vitro* studies may also provide evidence for the role of individual nuclei within multinucleated cells as the process of this specialization is poorly understood.

### Heterogeneous Myofiber Nuclei in Homeostatic Myofibers

**FIGURE 2:**
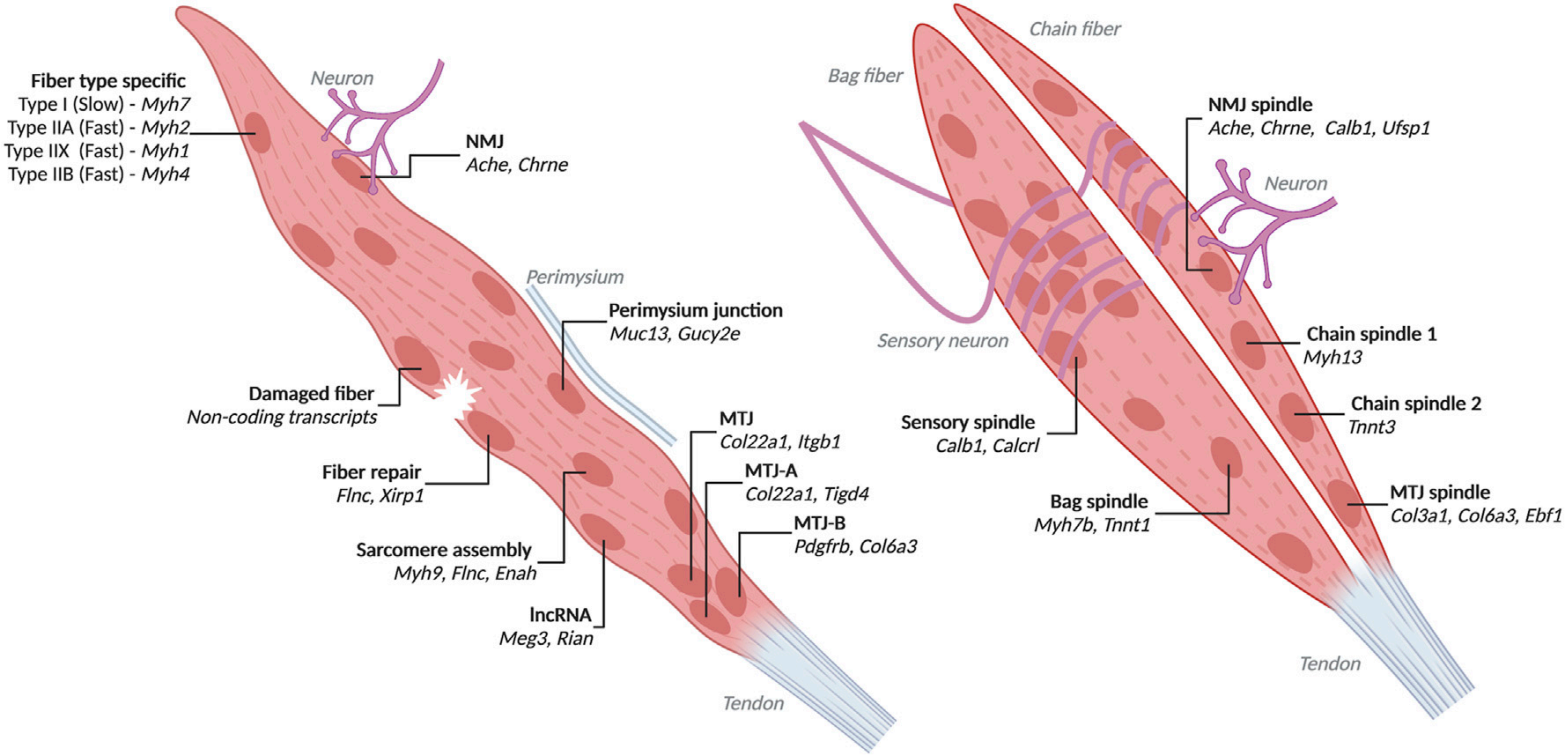
Heterogenous myofiber nuclei with associated markers identified by high-throughput transcriptomics Populations and top markers of nuclei from myofibers identified through single-nucleus RNA-seq and low throughput spatial transcriptomics. Left is a generic myofiber with transcriptionally distinct nuclei labelled. All populations except fiber type specific, sarcomere assembly and lncRNA are influenced by their proximity to other cells, damage or the perimysium. Right are bag and chain fibers. Created with BioRender.com.

Single-cell sequencing of mature muscle cells is low throughput as myofibers need to be isolated by hand. However, scRNA-seq of myofibers was able to parse gene expression arising specifically from myofibers as opposed to other cell types in the muscle tissue, such as the satellite cell marker *Pax7* and the fibroblast marker *Col1a1* (Blackburn et al., 2019). In the past 2 years, snRNA-seq has been applied to myofibers enabling discoveries into myofiber type and intranuclear heterogeneity. Muscle groups in mice and humans are made up of different types of muscle fibers, generally called fast and slow twitch. Each of these function in a slightly different way and express distinct genes and transcripts. Fast twitch fibers in mice and humans generally express *MYH2* (Type 2A), *MYH1* (Type 2X), *MYH4* (Type 2B) while slow fibers express *MYH7* (Figure 2) (Schiaffino and Reggiani, 2011). Single-nucleus RNA-seq on muscle tissue have recovered myonuclei from each of these fiber types expressing the respective *Myh* (Dos Santos et al., 2020; Kim et al., 2020; Petrany et al., 2020; Orchard et al., 2021). Nuclei in mice expressing different isoforms of *Myh4*, A, B or C, have distinct expression profiles and are found in differing proportions in different muscle groups (Dos Santos et al., 2020). Most nuclei express one *Myh* gene, as confirmed by RNAscope (Dos Santos et al., 2020). Nuclei expressing *Myh1* and *Myh2* are transcriptionally similar and are occasionally expressed in the same nucleus and often from the same allele (Dos Santos et al., 2020; Kim et al., 2020). These nuclei are mostly limited to the soleus whereas the majority of EDL (extensor digitorum longus) myonuclei express only one *Myh* (Dos Santos et al., 2020). The expression of *Myh* genes is coordinated in nuclei across the length of the myofiber in quadricep and EDL, but not in soleus (Dos Santos et al., 2020). Innervation by motor neurons is required for coordinated *Myh* expression, and this coordination is activated in early postnatal development (Dos Santos et al., 2020).

Myofibers in mice and humans interact with other cell types within the muscle tissue, notably neurons and tendons. Nuclei under NMJ and MTJ are transcriptionally distinct. NMJ nuclei express *Ache* (acetylcholinesterase) which hydrolyzes acetylcholine from neurons (Jacobson et al., 2001). MTJ nuclei are known to express the collagen *Col22a1* (Charvet et al., 2013). Single-nucleus RNA-seq in mice has revealed additional markers for these populations such as *Etv5*, *Etv4*, *Chrne*, *Colq*, *Musk*, *Ufsp1*, *Lrfn5*, *Ano4*, *Vav3* for NMJ nuclei and *Maml2*, *Ankrd1*, *Slc24a2*, *Adamts20* for MTJ nuclei (Petrany et al., 2020; Dos Santos et al., 2020) (Supplementary Table S2 Nuclei Populations). Additionally, *Ufsp1* and *Gramd1b* were found to regulate the specification of NMJ nuclei (Petrany et al., 2020). MTJ nuclei are also heterogeneous. MTJ nuclei expressing *Tigd4*, *Itgb1*, *Col24a1* and *Col22a1* were present in every fiber from adult mouse tibialis anterior. Only some fibers had MTJ nuclei which express *Pdgfrb*, *Ebf1*, *Col1a2*, *Col6a1*, and *Col6a3* (Kim et al., 2020). Overall, NMJ nuclei make up 0.8% of the myonuclei from adult mouse tibialis anterior, while MTJ nuclei are about 3.6% (Petrany et al., 2020).

Skeletal muscles in mice and humans contain spindle fibers, which are composed of intrafusal fibers, innervated by sensory neurons that are responsible for proprioception (Kröger and Watkins, 2021). Spindle fiber nuclei can be marked by expression of *Calb1* (Kim et al., 2020). Spindle fibers are classified as either bag or chain fibers based on the arrangement of nuclei in the cell (Kröger and Watkins, 2021). Bag fiber nuclei express *Myh7b* and *Tnnt1*, while chain fiber nuclei are heterogeneous with expression of either *Myh13* or *Tnnt3* (Kim et al., 2020). Spindle fibers also contain NMJ nuclei under motor neurons and MTJ nuclei. Areas in contact with sensory neurons contain densely packed nuclei which express *Calcrl* and are distinct from nuclei innervated by motor neurons (Kim et al., 2020).

Interestingly, some transcriptionally distinct nuclei in mice express lncRNAs from the *Dlk1-Dio3* locus, specifically *Rian* and *Meg3* (Kim et al., 2020; Petrany et al., 2020). RNA FISH for *Rian* found that these nuclei are dispersed throughout myofibers (Kim et al., 2020). Found on the outer edge of fibers near the perimysium are nuclei expressing *Muc14* and *Gucy2e* which may be specialized to help in adhesion (Kim et al., 2020). Additional populations of heterogeneous nuclei have been identified but poorly described with expression of *Gssos2*, *Suz12* or *Bcl2* and possibly relating to the ER, epigenome or steroid synthesis, respectively (Kim et al., 2020).

The nuclei populations identified inform our understanding of basic muscle biology. Some populations, such as NMJ or MTJ, are clearly defined by proximity to other structures that inform the role they play within the myofiber. Other nuclei, such as those marked by lncRNA expression, have unknown roles and etiology. More work is needed to understand the complex specification and interaction of transcriptionally distinct nuclei within the same cell.

Current snRNA-seq studies in myofibers have only been done on mouse. Studies in human biopsies could reveal new nuclear heterogeneity specific to humans. Additionally, skeletal muscle groups throughout the body have different fiber type compositions and other differences that could be surveyed using snRNA-seq. For example, Dos Santos, et al. found different levels of *Myh* co-expression in the soleus than in the EDL (Dos Santos et al., 2020). Muscular dystrophies often affect some muscle groups more severely than others. Thus, snRNA-seq analyses in multiple muscle groups may reveal factors contributing to susceptibility.

### Skeletal Muscle Cell and Nucleus Populations Identified in Aging Tissue

During aging in mice and humans, muscle mass is lost and not regenerated leading to loss in strength (Naranjo et al., 2017). Aged satellite cells in humans have reduced activation and therefore cannot restore lost fibers or the satellite cell pool (Carlson et al., 2009). In spite of this, aged satellite cells are also more likely to exit quiescence in mice (Hernando-Herraez et al., 2019; Shcherbina et al., 2020). Retinoic acid receptors help to maintain satellite cell quiescence but are lost with age in mice (Shcherbina et al., 2020). The aged satellite cells in mice follow the normal regeneration trajectory but are delayed in activation (Kimmel et al., 2020). Upon activation, they upregulate genes related to stress, inflammation and immune response (Kimmel et al., 2020; Shcherbina et al., 2020). Transcription in aged satellite cells is uncoordinated possibly due to stochastic methylation differences between aged cells (Hernando-Herraez et al., 2019). The variability in expression between cells leads to dysregulation of genes for interaction with the cell-niche (Hernando-Herraez et al., 2019).

Comparison of whole myofibers isolated from old and young mice identified dysregulation of genes related to muscle growth and structure, such as *Actc1* and *Myl1*, collagen synthesis and metabolism that may contribute to age related muscle function decline (Blackburn et al., 2019). The sarcomere assembly population present abundantly in P21 mice are also present in aged mice (Petrany et al., 2020). A subset of aged nuclei express *Ampd3* as well as genes for immune response and apoptosis (Petrany et al., 2020). These nuclei may represent dysfunction due to denervation (Petrany et al., 2020). Understanding cellular heterogeneity in aged muscle revealed potential contributors to sarcopenia, while the contribution of age-specific myofiber nuclei populations is unclear.

### Use of High-Throughput and High-Resolution Transcriptome Methods to Study Muscular Dystrophies

#### Facioscapulohumeral Muscular Dystrophy

Cellular heterogeneity is known to play an important role in some disease contexts, such as facioscapulohumeral muscular dystrophy (FSHD). FSHD is linked to the misexpression of an embryonic transcription factor, *DUX4* (Lemmers et al., 2010; Vanderplanck et al., 2011). DUX4 misexpression causes downstream dysregulation of embryonic genes and retrotransposons such as ERVLs (Vanderplanck et al., 2011; Geng et al., 2012; Young et al., 2013; Knopp et al., 2016). Previous studies have sought to identify the patient-specific transcriptome using bulk RNA-seq using multiple cells (Yao et al., 2014; Rickard et al., 2015). However, *DUX4* is rarely detected at the protein or RNA level in patient muscle (0.5% of patient myotube nuclei) (Tassin et al., 2013). Bulk RNA-seq averages out the signal from the few muscle cells that express *DUX4*. Thus, scRNA-seq and snRNA-seq have been particularly useful in looking at native expression of *DUX4* and its relationship with downstream target genes and comparing gene alterations in DUX4-expressing and non-expressing patient muscle cells.

With scRNA-seq on fusion-inhibited 72 h differentiated cells, between 0.2 and 0.9% of FSHD cells were found to express *DUX4,* higher than previously reported (van den Heuvel et al., 2019; Rickard et al., 2015). *DUX4* expression has been suggested to be burst-like and to cause immediate cell death, which may account for its rare detection (Feng et al., 2015; Rickard et al., 2015). DUX4 target genes were more readily detectable in cells than *DUX4* which may suggest a transient burst of *DUX4* expression followed by more sustained activation of downstream pathways (van den Heuvel et al., 2019). Comparison of these affected cells to other non-affected FSHD cells identified dysregulation of transcriptional regulators and confirmed the dysregulation of pathways previously identified to be affected by DUX4 from bulk RNA-seq studies (Yao et al., 2014; Rickard et al., 2015). scRNA-seq enabled identification of transcriptional regulators that could activate *DUX4* expression or aid in gene dysregulation following DUX4 expression.

With snRNA-seq of native multinucleated FSHD patient myotubes, *DUX4* transcript was detected in 0.1% of nuclei, which is much higher than the results of fusion-inhibited scRNA-seq (Jiang et al., 2020). Interestingly, RNAScope detection of *DUX4* transcripts revealed accumulation in the nucleus, suggesting that sequencing only the nuclear RNA might have made it easier to detect *DUX4* expression (Jiang et al., 2020; Chau et al., 2021). Nuclei expressing DUX4 targets made up 3.7% which is much higher than *DUX4*-expressing nuclei (Jiang et al., 2020). This may be the result of spreading of DUX4 protein to multiple nuclei in the same myotube to activate target genes (Tassin et al., 2013; Rickard et al., 2015; Chau et al., 2021). However, *in situ* RNA detection revealed the expression of target genes without detectable DUX4 transcript or protein in some of the patient myotubes, raising the possibility that, once activated, target gene expression may be maintained in these myotubes even in the absence of DUX4 (Jiang et al., 2020; Chau et al., 2021). The *DUX4* homolog and target gene, *DUXA*, is expressed in many more nuclei than *DUX4* and can regulate the expression of at least two DUX4 target genes (Jiang et al., 2020). This indicates that DUX4 target genes themselves participate in the maintenance of the DUX4 gene network (Jiang et al., 2020). Additionally, two populations of FSHD nuclei are apparent with high (FSHD-Hi) or low (FSHD-Lo) DUX4 target gene expression (Jiang et al., 2020). The FSHD-Hi nuclei appear to inappropriately activate cell cycle genes despite the myotubes having entered G0 (Jiang et al., 2020). The FSHD-Lo transcriptome is distinct from that of control nuclei, suggesting that patient cells are altered even in the absence of DUX4 and target gene expression (van den Heuvel et al., 2019; Jiang et al., 2020). The identification of these populations of nuclei and the precise co-expression of *DUX4* with its target genes in native, fused myotube nuclei was only possible with nuclear resolution afforded by snRNA-seq and spatial transcriptomics. Overall, the use of single-cell and single-nucleus RNA-seq in conjunction with *in situ* RNA FISH helped identify pathogenic populations of nuclei that express *DUX4* and the factors contributing to its expression and disease progression.

#### Duchenne Muscular Dystrophy

Duchenne muscular dystrophy is the most common form of muscular dystrophy arising from nonsense mutations in the structural protein dystrophin leading to its loss of function (Gao and McNally, 2015; Wilson et al., 2017). The altered dystrophin mainly affects myofibers in which dystrophin connects the center of the cell to the membrane (Gao and McNally, 2015). The MDX mouse model of DMD produces a truncated form of dystrophin and is a popular model for studying DMD (McGreevy et al., 2015). This model provides a way to assess the disease pathology in active muscle which includes cell death and muscle repair (Chang et al., 2016; Morgan et al., 2018; Sreenivasan et al., 2020). Accordingly, snRNA-seq and RNAscope of MDX myofibers found that a subset of nuclei near sites of damage appear to activate repair with co-expression of *Flnc* and *Xirp1* (Kim et al., 2020). Notably, these nuclei appear similar to nuclei identified previously in P21 mice and aged muscle and are thought to be involved in sarcomere assembly (Petrany et al., 2020). This subset is also observed in biopsies from DMD and patients with mutations in dysferlin which plays a role in myofiber membrane repair (Bansal et al., 2003; Kim et al., 2020). MDX myofibers also have nuclei that are associated with dying myofibers with leaky membranes (Kim et al., 2020). These nuclei express an abundance of noncoding transcripts, and are in close proximity to infiltrating cells, thought to be macrophages (Kim et al., 2020). Macrophage infiltration into skeletal muscle can exacerbate DMD (Kharraz et al., 2013). Interestingly, MDX myofibers lack substantial NMJ nuclei which is consistent with the disruption of NMJ structure in the MDX mouse model (Kim et al., 2020).

A second mouse model of DMD with exon 51 of *DMD* deleted (ΔEx51) shows the greatest transcriptional alterations in the NMJ and MTJ nuclei out of all nuclei (Chemello et al., 2020). All ΔEx51 nuclear populations upregulated genes involved in the ubiquitin-proteasome system. MTJ nuclei upregulated *Foxo3* which activates the ubiquitin-proteasome system in myofibers and has been shown to induce atrophy *in vitro* myotubes (Chemello et al., 2020). Populations of nuclei expressed distinct apoptotic markers. Expression of *p38* in MTJ and NMJ nuclei supports a distinct role for these nuclei in disease etiology (Chemello et al., 2020). A population of regenerative myonuclei which express markers of differentiation and fusion were found specifically in the ΔEx51 mice. *Jdp2* expression may regulate expression of genes specific to these nuclei and therefore play a role in regulating regenerative nuclei (Chemello et al., 2020).

The identification of apparently dystrophic specific nuclear populations holds promise for future investigation into their roles in pathogenesis. These subcellular signatures provide biomarkers for active disease that are otherwise not observable at the whole cell level. Understanding that multinucleated cells contain disease associated specialized myonuclei has important implications for conclusions drawn from bulk RNA-seq studies as signatures of these nuclei are lost. Additionally, single-cell RNA-seq on mononucleated cells from disease models or patients is not sufficient to fully understand disease pathology in myofibers. Due to heterogeneity of the nuclei, high-resolution transcriptome assays in skeletal muscle should be performed with nucleus level resolution.

### Future Prospects

Single nucleus resolution transcriptome methods in muscle have the advantage of being able to answer fundamental questions about skeletal muscle that are unanswerable by other methods. Muscle development is known to be mutually reliant on tenocyte development with mechanical stress stimulating differentiation (Subramanian and Schilling, 2015; Valdivia et al., 2017). High-resolution transcriptomic methods can help reveal how mechanical force is translated to gene expression changes in specific nuclei to stimulate maturation.

Disorders such as sarcopenia, FSHD and DMD affect the transcriptomes of subsets of cells and nuclei that are important for understanding pathogenesis (Hernando-Herraez et al., 2019; Jiang et al., 2020; Kim et al., 2020). Additional neuromuscular diseases are known to affect myofiber nuclei. Myofibers from spinal muscular atrophy (SMA) have centrally localized nuclei and loss of innervation (Dastur and Razzak, 1973). Understanding how denervation affects subpopulations of nuclei, such as in the NMJ, in SMA would require single nucleus resolution techniques (Swoboda et al., 2005).

To address the mechanism of nucleus specialization, single nucleus transcriptome methods need to be combined with additional assays. Assays for single-cell ATAC-seq, DNA methylation sequencing and ChIP-seq are already available, and some of these assays can be combined with RNA-seq from the same cell or nucleus (Smallwood et al., 2014; Clark et al., 2016; Clark et al., 2018; Grosselin et al., 2019; Lareau et al., 2019; Linker et al., 2019). Single-nucleus ATAC-seq on myofiber nuclei has already identified potential transcriptional regulators of fiber type (Dos Santos et al., 2020), and multiomic measurements of RNA and chromatin accessibility from the same nuclei will likely further clarify the regulation of myonuclear states. Single-cell DNA methylation has identified stochastic methylation changes which result in alterations to transcriptional networks in aged muscle cells (Hernando-Herraez et al., 2019). Using additional high-resolution methods in combination with snRNA-seq can help to identify heterogenous gene regulatory networks acting across nuclei within the same cell.

Understanding transcriptional heterogeneity of myonuclei enables us to understand the role these nuclei play within the myofiber. The role of nuclei populations is important in understanding underlying mechanisms for diseases which affect specific populations, such as the NMJ in DMD (Ng and Ljubicic, 2020). Ablation of specific populations through factors which regulate heterogeneity would help us understand if nuclear heterogeneity is required for homeostatic function and how nuclei populations impact nuclei in the same cell.

## Conclusion

Skeletal muscle is composed of heterogeneous mononuclear cells and myofibers with transcriptionally specialized nuclei. This level of diversity is finally being discovered using high-throughput, high-resolution transcriptome studies. Mononucleated cells are mostly heterogeneous due to their state in myogenesis, and sarcopenia appears to affect activation of myogenesis. Upon differentiation *in vitro*, sets of cells do not fuse to form myotubes but instead remain mononucleated. Nuclei in fused myotubes begin to show specialization in *vitro* culture. Nuclei from mature myofibers show a high degree of specialization depending on fiber type and proximity to other cell types. However, not all nuclear heterogeneity is attributable to these differences. Sets of nuclei appear to be transcriptionally distinct with specific sets of marker genes such as *Flnc* or lncRNAs. Classifying diverse nuclei types is the first step in understanding how these populations contribute to muscle biology. By looking in perturbed or disease systems which lose or alter specific nuclei populations, we can start to understand the role of these nuclei within the myofiber. Diseases such as FSHD and DMD also cause myofiber nuclei to specialize for example by activation of pathogenic genes or in response to damage. These studies have only begun to unlock the heterogeneity of myonuclei and to demonstrate the importance of surveying myofibers at the level of the nucleus.

## Supplementary Material

Supplementary Table 1 Populations of Heterogeneous Mononuclear Cells and Myotube Nuclei.

Supplementary Table 2 Populations of Heterogeneous Myofiber Nuclei.

## Abbreviations

DMD, Duchenne muscular dystrophy; EDL, extensor digitorum longus; FACS, fluorescence-activated cell sorting; FAP, fibroadipogenic progenitor; FSHD, Facioscapulohumeral muscular dystrophy; IMFs, interstitial muscle fibroblasts; iPSCs, induced pluripotent stem cells; ITS, insulin-transferrin-selectin; lncRNA, long non-coding RNA; MERFISH, multiplexed error-robust fluorescence *in situ* hybridization; MRF, myogenic regulatory factor; MTJ, myotendinous junction; NMJ, neuromuscular junction; RNA FISH, RNA fluorescent *in situ* hybridization; RNA-seq, RNA sequencing; scRNA-seq, single-cell RNA sequencing; SMA, spinal muscular atrophy; snRNA-seq, single-nucleus RNA sequencing.
