# Supplementary material for: Heterogeneous Skeletal Muscle Cell and Nucleus Populations Identified by Single-Cell and Single-Nucleus Resolution Transcriptome Assays
Source: Front Genet. 2022 May 13;13:835099. doi: 10.3389/fgene.2022.835099 (PMC9136090; doi:10.3389/fgene.2022.835099)
Supplement: Supplementary file 1 [file Table1.docx]

**Table S1: Populations of Heterogeneous Mononuclear Cells and Myotube Nuclei**

|  | **Top Markers** | **References** |
| --- | --- | --- |
| ***in vitro* Populations** |  |  |
| Myoblast | *Myod1, Myf5* | [42] |
| MNCs | *Id1, Id3, Pdgfra, Sphk1* | [42,63] |
| ECM remodeling | *Col1a1, Fn1* | [64] |
| Activated satellite cell-like | *Itm2a, Pax7* | [64] |
| Myotube nucleus | *Myog, Myh3* | [42,63] |
| *Myog*+ | *Myog*, *Mef2a* | [64] |
| *Mef2c*+ | *Mef2c* | [64] |
|  |  |  |
| **Development Populations** |  |  |
| Adult satellite cell precursor | *Pax7, Msc* | [57] |
| IMF precursor | *Col1a1, Osr1/2, Myod1, Myog* | [57] |
| Mature myocyte | *Tnnc* | [57] |
|  |  |  |
| ***in vivo* Populations** |  |  |
| Quiescent satellite cell | *Pax7, Btg* | [34,58] |
| Human specific quiescent satellite cell | *CAV1, SPRY1, HEY1* | [62] |
| Activated satellite cell | *Pax7, Myod1* | [60] |
| Myoblast | *Myod1, Myf5* | ﻿[24,34] |
| Cycling myoblast | *Ccnd1/2, Ccnb2* | [34,58,59] |
| Differentiating myoblast | *Myog, Tnnt2* | [34,58,59] |
| Myofiber associated satellite cell | *Pax7, Myf5, Sdc4* | [51] |
