# Supplementary material for: Heterogeneous Skeletal Muscle Cell and Nucleus Populations Identified by Single-Cell and Single-Nucleus Resolution Transcriptome Assays
Source: Front Genet. 2022 May 13;13:835099. doi: 10.3389/fgene.2022.835099 (PMC9136090; doi:10.3389/fgene.2022.835099)
Supplement: Supplementary file 2 [file Table2.docx]

**Table S2: Populations of Heterogeneous Myofiber Nuclei**

|  | **Top Markers** | **Additional Markers** | **References** |
| --- | --- | --- | --- |
| Fiber Type Specific Populations |  |  |  |
| Type I (Slow) | *Myh7* | NA | [44] |
| Type IIA (Fast) | *Myh2* | NA | [44] |
| Type IIX (Fast) | *Myh1* | NA | [44] |
| Type IIB (Fast) | *Myh4* | NA | [44] |
|  |  |  |  |
| Subcellular Localized Populations |  |  |  |
| NMJ | *Ache, Chrne* | *Etv5, Musk, Lrp4, Colq, Chrna1, Prkar1a, Etv4, Ufsp1, Lrfn5, Ano4, Vav3, Ablim2, Phldb2, Irf8* | [43–45] |
| MTJ | *Col22a1, Itgb1* | *Slc24a2, Adamts20, Ankrd1, Maml2, Col24a1, Tigd4, Col1a2, Col6a1, Col6a3, Pdgfrb, Ebf1* | [43–45] |
| MTJ-A | *Tigd4, Col22a1* | *Itgb1, Col24a1, Smad3* | [45] |
| MTJ-B | *Pdgfrb, Col6a3* | *Ebf1, Col1a2, Col6a1* | [45] |
| Perimysium junction | *Muc13, Gucy2e* | *NA* | [45] |
|  |  |  |  |
| Homeostatic Populations |  |  |  |
| Sarcomere assembly | *Myh9, Flnc, Enah* | *Runx1, Nrap, Fhod3, Myh10, Ifrd1, Nfat5, Mef2a, Ell, Creb5, Zfp697, Atf3* | [43,44] |
| lncRNA | *Meg3, Rian* | *Mirg* | [43,45] |
|  |  |  |  |
| Damage Associated Populations |  |  |  |
| Damaged fibers | *Gm10801, Gm10717* | NA | [45] |
| Fiber repair | *Flnc, Xirp1* | NA | [45] |
|  |  |  |  |
| Spindle Fiber Populations |  |  |  |
| Bag spindle | *Myh7b* | *Tnnt1, Piezo2* | [45] |
| Chain spindle 1 | *Myh13* | NA | [45] |
| Chain spindle 2 | *Tnnt3* | NA | [45] |
| NMJ spindle | *Chrne, Ache, Calb1* | *Ufsp1, Piezo2* | [45] |
| MTJ spindle | *Col3a1, Col6a3, Ebf1* | *Calb1, Col6a1* | [45] |
| Sensory spindle | *Calb1, Calcrl* | NA | [45] |
